# Huangjia Ruangan Granule Inhibits Inflammation in a Rat Model with Liver Fibrosis by Regulating TNF/MAPK and NF-*κ*B Signaling Pathways

**DOI:** 10.1155/2022/8105306

**Published:** 2022-07-30

**Authors:** Qiang Cai, Zongquan Wang, Rong Zhang, Lili Zhang, Sainan Cui, Huiyuan Lin, Xinran Tang, Dongying Yang, Xianrong Lin, Shasha Bai, Jin Gao, Lei Yang

**Affiliations:** ^1^School of Pharmaceutical Sciences, Guangzhou University of Chinese Medicine, Guangzhou 510000, China; ^2^Yingkerui (Hengqin) Pharmaceutical Research Institute Co., Ltd, Zhuhai 519000, China

## Abstract

The Huangjia Ruangan granule (HJRG) is a clinically effective Kampo formula, which has a significant effect on liver fibrosis and early liver cirrhosis. However, the mechanism underlying HJRG in treating liver fibrosis remains unclear. In this study, carbon tetrachloride (CCl_4_) was used to induce liver fibrosis in rats to clarify the effect of HJRG on liver fibrosis and its mechanism. Using network pharmacology, the potential mechanism of HJRG was initially explored, and a variety of analyses were performed to verify this mechanism. In the liver fibrosis model, treatment with HJRG can maintain the liver morphology, lower the levels of AST and ALT in the serum, and ameliorate pathological damage. Histopathological examinations revealed that the liver structure was significantly improved and fibrotic changes were alleviated. It can effectively inhibit collagen deposition and the expression of *α*-SMA, reduce the levels of the rat serum (HA, LN, PC III, and Col IV), and inhibit the expression of desmin, vimentin, and HYP content in the liver. Analyzing the results of network pharmacology, the oxidative stress, inflammation, and the related pathways (primarily the TNF signaling pathway) were identified as the potential mechanism of HJRG against liver fibrosis. Experiments confirmed that HJRG can significantly increase the content of superoxide dismutase and glutathione and reduce the levels of malondialdehyde and myeloperoxidase in the rat liver; in addition, HJRG significantly inhibited the content of proinflammatory cytokines (TNF-*α*, IL-1*β*, and IL-6) and reduced the expression of inflammatory regulators (Cox2 and iNOS). Meanwhile, treatment with HJRG inhibited the phosphorylation of NF-*κ*B P65, I*κ*B*α*, ERK, JNK, and MAPK P38. Moreover, HJRG treatment reversed the increased expression of TNFR1. The Huangjia Ruangan granule can effectively inhibit liver fibrosis through antioxidation, suppressing liver inflammation by regulating the TNF/MAPK and NF-*κ*B signaling pathways, thereby preventing the effect of liver fibrosis.

## 1. Introduction

Liver fibrosis is a pathophysiological process. It is the abnormal proliferation of connective tissue in the liver caused by various pathogenic factors, which is a serious health problem for humans. Without intervention and treatment, liver fibrosis may progress into liver cirrhosis or even liver cancer [1]. At present, the treatment of liver fibrosis is primarily based on the original cause of the disease, such as anti-inflammatory therapy [2], regulating the body's oxidative stress [3], inhibiting the deposition of the extracellular matrix (ECM) in the liver, and promoting ECM degradation [4]. Clinical studies have shown that liver fibrosis can be reversed. Therefore, in the stage of liver fibrosis or even the advanced stage of liver fibrosis, taking preventive interventions can effectively improve the clinical results [5].

CCl_4_ is a common hepatotoxin, widely used to induce toxic effects in the liver of experimental animal models, which can induce liver inflammation and fibrosis in rats [6]. Prolonged exposure to CCl_4_ can promote lipid peroxidation, and reactive oxygen species (ROS) are excessively produced in the body, leading to the consumption of glutathione (GSH), superoxide dismutase (SOD), malondialdehyde (MDA), and myeloperoxidase (MPO) formation, which causes oxidative stress [7]. The induction of CCl_4_ further lures inflammation in the liver tissues, leading to the release of various inflammatory cytokines, such as tumor necrosis factor-alpha (TNF-*α*, IL-6, and IL-1*β*), and inflammatory regulators (Cox2 and iNOS), thus causing hepatitis [8]. Moreover, the increase of inflammatory cytokines leads to further aggravation in the oxidative stress, thereby maintaining a vicious circle, leading to the conversion of HSCs from a resting state to myofibroblast-like cells, then activating the myofibroblasts, which are responsible for matrix remodeling and the increasing levels of ECM proteins such as collagen, desmin, vimentin, and laminin [9], thereby leading to liver fibrosis, liver cirrhosis, and liver cancer. Therefore, medical interventions that are aimed at blocking oxidative stress and inflammation are crucial in reversing liver fibrosis.

Transcriptional regulatory nuclear factor (NF-*κ*B P65) is an important mediator of the inflammatory signal to stimulation [10]. A large number of studies have shown that the upregulation of NF-*κ*B P65 can stimulate the proliferation of hepatic stellate cells (HSCs) and inhibit HSC apoptosis, which plays a key role in fibrosis [11]. Meanwhile, the MAPK pathway is another major extracellular signal transduction pathway stimulated by inflammatory mediators. The MAPK family includes three components: c-Jun N-terminal kinase (JNK), extracellular signal receptor-activated kinase (ERK) 1/2, and p38 MAPK. Once stimulated by signals, ERK/JNK/p38 is phosphorylated to mediate inflammatory responses [12]. TNFR1 is thought to be the major receptor involved in the activation of the NF-*κ*B and MAPK pathways, which in turn induces the expression of proteins of the inflammatory response and plays an important role in regulating the inflammatory response [13].

The Huangjia Ruangan granule is a pure Chinese medicine compound preparation, which is composed of *Hedysarum Multijugum Maxim* (Huangqi, HQ), *Trionyx sinensis Wiegmann* (Biejia, BJ), *Radix Puerariae* (Gegen, GG), *Radix Bupleuri* (Chaihu, CH), *Ganoderma* (Lingzhi, LZ), *Radix Paeoniae Rubra* (Chishao, CS), *Radix Salviae* (Danshen, DS), *Panax Notoginseng* (Sanqi, SQ), *Abri Herba* (Jigucao, JGC), and *Phyllanthus urinaria* L (YeXiaZhu, YXZ). HQ and CH are used to ameliorate liver injury [14, 15]; BJ can soften and disperse knots; LZ, CS, DS, and SQ can promote the blood circulation and have antioxidant and anti-inflammatory effects [16–19]; GG, JGC, and YXZ can detoxify damp-heat residual toxins [5, 20, 21]. The combination of these drugs exerts a variety of pharmacological effects, such as antioxidation, anti-inflammatory, and antifibrosis while protecting the liver and relieving depression. In addition, it is clinically used to relieve liver fibrosis and early liver cirrhosis. However, the specific mechanism of how HJRG relieves liver fibrosis remains unclear.

Chinese medicine has attracted great interest in the treatment of liver fibrosis for its efficacy and few side effects [22], but its chemical composition is complex, and it acts on multiple targets in different ways. All of these pose challenges to the understanding of underlying mechanisms [23]. Network pharmacology is based on system biology and network analysis [24]. Given the rapid development of various bioinformatics resources, network pharmacology methods have been applied to the discovery of active ingredients and molecular mechanisms of Chinese medicine [25]. Therefore, the research of Chinese medicine based on network pharmacology is worth exploring.

We first studied the pharmacodynamics of rats with liver fibrosis, and results have showed that HJRG demonstrated obvious antiliver fibrosis effects. Afterward, we searched the chemical composition and targets of HJRG through the database and then constructed a composite target-disease network combined with relevant literature to predict the mechanism of HJRG in the treatment of liver fibrosis. Finally, we verified its potential mechanism through pharmacological experiments and clarified that the Huangjia Ruangan granule can effectively inhibit liver fibrosis through antioxidation, inhibiting liver inflammation by regulating the TNF/MAPK and NF-*κ*B signaling pathways, thereby preventing the effect of liver fibrosis.

## 2. Materials and Methods

### 2.1. Animals and Experimental Design

A total of 72 male SD rats, specific pathogen-free (SPF) grade, weighing 180–220 g, were provided by the Laboratory Animal Center of Guangzhou University of Traditional Chinese Medicine (experimental animal use license number: SYXK (Guangdong) 2018–0085; Laboratory animal production license number: SCXK (Guangdong) 2018-0034). The experimental rats were kept in an SPF animal room with suitable standard environmental temperature and humidity. During the breeding period, the rats were exposed to sunlight for 12 h every day. After 1 week, the rats were randomly divided into six groups: the control group (Control), the model group (Model), the silymarin group (Silymarin), the Huangjia Ruangan granule low-dose group (HJRG-L), the Huangjia Ruangan granule medium-dose group (HJRG-M), and the Huangjia Ruangan granule high-dose group (HJRG-H). Twelve rats were included in each group. The control group was given the corresponding olive oil two times a week for 10 weeks to induce liver fibrosis in the rat models, whereas the other groups were given a 20% CCl_4_ olive oil solution (3 mL/kg) by gavage. From the second day of modeling, the administration group was given the corresponding test drug by gavage. The silymarin group was given the silymarin solution at a daily dose of 25 mg/kg/d. The Huangjia Ruangan granule low-dose group (1.16 g/kg/d), the middle-dose group (2.32 g/kg/d), and the high-dose group (4.64 g/kg/d) were given a daily dose of the Huangjia Ruangan granule solution by gavage, and the control group and the model group were given the corresponding amount of drinking water by gavage once a day for 10 weeks. Silymarin was purchased from Shanghai Macklin Biochemical Co., Ltd (Shanghai, China, cat no.S873807), and the Huangjia Ruangan granule was obtained from YingKeRui (Hengqin) Pharmaceutical Research Institute Co., Ltd (Zhuhai, China, cat no. 20190615)

### 2.2. HPLC Analysis

**Figure 1:**
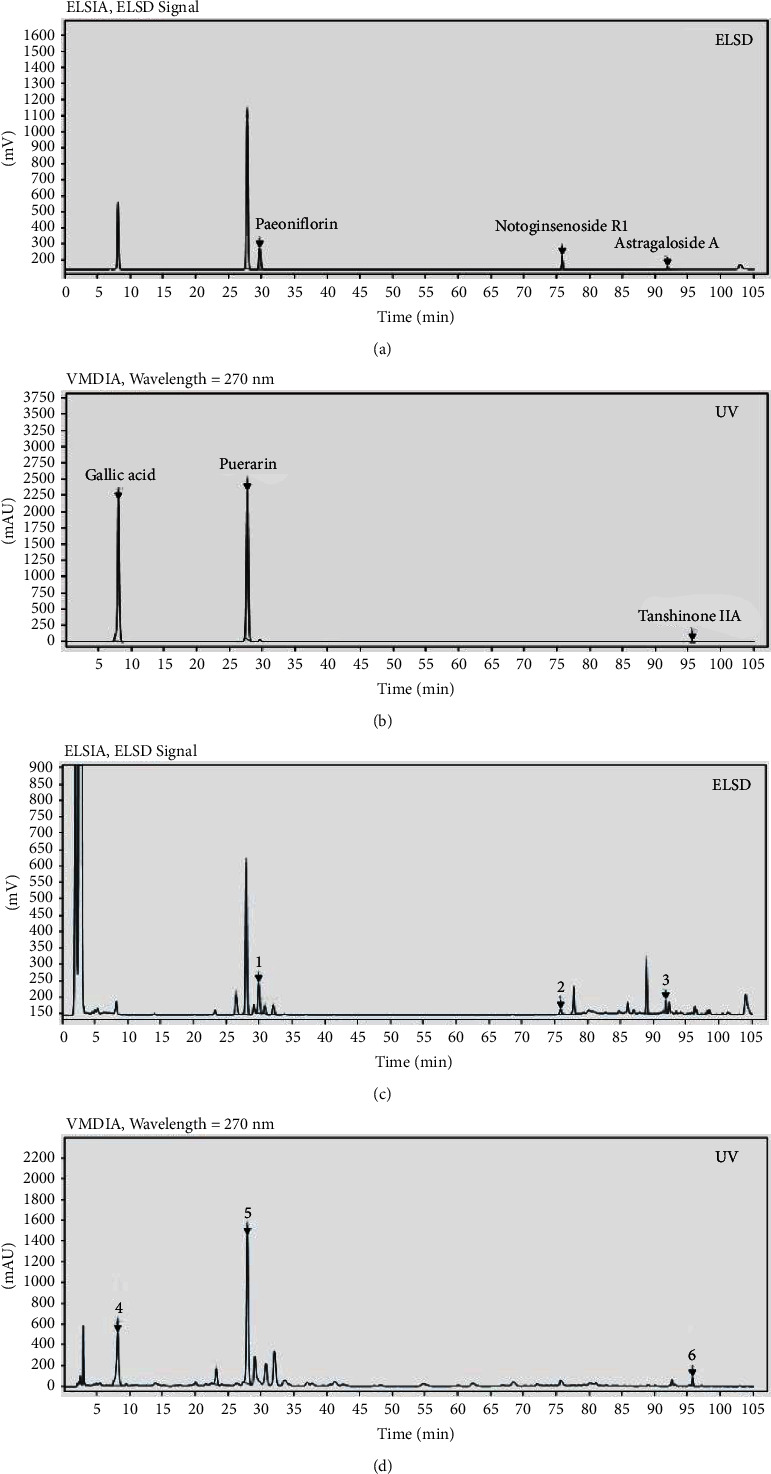
The results of HJRG by HPLC. (a) The reference substance of paeoniflorin, notoginsenoside R1, and astragaloside A in HPLC. (b) The reference substance of gallic acid, puerarin, and tanshinone IIA in HPLC. (c) The HPLC analysis results of HJRG. (1) Paeoniflorin; (2) notoginsenoside R1; (3) astragaloside (A). (d) The HPLC analysis results of HJRG. (4) Gallic acid. (5) Puerarin. (6) Tanshinone IIA.

A HPLC assay was carried out using a Waters Symmetry-C18 column (250 mm × 4.6 mm, 5 *μ*m). The phases were mobile phase *A* = methanol and mobile phase *B* = 0.1% aqueous acetic acid. Program gradient: 0–25 min, 5%–30% A; 25–50 min, 30%–35% A; 50–65 min, 35%–45% A; 65–95 min, 45%–95% A; 95–105 min, 95% A. The flow rate was 1 mL/min. The ELSD was used for paeoniflorin, notoginsenoside R1, and astragaloside. The detection wavelength was set at 270 and was used for gallic acid, puerarin, and tanshinone IIA (Figure 1).

### 2.3. Histopathological Analysis of the Liver

After fixing the liver with 4% paraformaldehyde, it was dehydrated with ethanol, transparent with xylene, and embedded in paraffin to prepare 4 *μ*m-thick histopathological sections for eosin (H&E) staining (Beyotime Bio, cat no. C0105) and Sirius red staining (Legend Bio, cat no. DC0041) to observe histopathology and collagen formation. Sirius red staining was quantified by the Image J software.

### 2.4. Serum Biochemical Analysis and Hepatic HYP Content Analysis

After the rats were anesthetized, blood was drawn from the abdominal aorta, and the serum was obtained by centrifugation. The operation was performed according to the kit instructions to determine the contents of ALT (Nanjing Jiancheng Bio, cat no. C009-2-1) and AST (Nanjing JianchengBio, cat no. C010-2-1) in the serum, and HYP (Nanjing Jiancheng Bio, cat no. A030-2-1) in the liver tissues. The serum PC III (JiangLai Bio, cat no. JL20799), Col-IV (JiangLai Bio, cat no. JL20754), HA (JiangLai Bio, cat no. JL10946), and LN (JiangLai Bio, cat no. JL13737) contents were determined by enzyme-linked immunosorbent assay (ELISA).

### 2.5. Network Pharmacological Analysis

The active ingredients of the Huangjia Ruangan granule and their corresponding targets were collected on the basis of the Traditional Chinese Medicine System Pharmacology Analysis Platform (TCMSP). Relevant target information of liver fibrosis was obtained from the OMIM database, TTD database, GeneCards database, and DisGeNet database. The intersection was used to obtain the predicted target of the Huangjia Ruangan granule for the treatment of liver fibrosis, and Cytoscape software was used to construct the “C (component)-T (target)” action network diagram. The DAVID database was used to enrich the targets in the network for GO enrichment and KEGG pathway enrichment, analyze the biological treatment process of the Huangjia Ruangan granule on liver fibrosis and the signal pathway conduction process, and then predict the drug mechanism. The enrichment analysis results were visualized through OmicShare.

### 2.6. Analysis of Oxidative Stress Indicators

The liver tissue was accurately weighed, and nine times the volume of normal saline was added to a weight (g)-to-volume (mL) ratio of 1 : 9. The liver was cut, and the homogenate was prepared in an ice water bath, centrifuged at 3000 rpm for 10 min, and collected. The supernatant was a 10% homogenized supernatant to be tested. According to the kit instructions, the expression levels of malondialdehyde (MDA) (Nanjing Jiancheng Bio, cat no. A003-1), GSH (Nanjing Jiancheng Bio, cat no. A006-2-1), and SOD (Nanjing Jiancheng Bio, cat no. A001-3) in the rat liver tissues were determined.

### 2.7. Myeloperoxidase (MPO) Activity

The liver tissue was accurately weighed. The second reagent solution prepared by using the kit as the homogenization medium was used, and the homogenization medium was added to a weight-to-volume ratio of 1 : 19 to prepare a 5% tissue homogenate without centrifugation. According to the kit instructions, the MPO (Nanjing Jiancheng Bio, cat no. A044-1-1) activity in the rat liver homogenate was determined.

### 2.8. Western Blot Analysis

The liver tissue was accurately weighed, and tissue lysate was added to a weight (mg)-to-volume (*μ*L) ratio of 1 : 10. Then, a tissue homogenate was prepared using a tissue homogenizer and centrifuged at 12000 rpm at 4°C for 15 min. The supernatant was collected, quantified using the BCA protein assay kit (Keygen Biotech), and boiled in a metal heater at 100°C for 10 min for denaturation. Approximately 50 mg of the total protein was separated by 12% SDS-polyacrylamide gel electrophoresis and transferred to a polyvinylidene fluoride membrane. The membrane was blocked with 5% skimmed milk powder for 2 h. The primary antibody was incubated at 4°C overnight (over 12 h), and then the corresponding secondary antibody was incubated on a shaker at room temperature for 1.5 h. The bound protein was detected using the Bio-Rad imaging system. The gray value of each band relative to the internal reference protein of the same sample was analyzed on the ImageJ software. The main antibodies detected by western blotting were as follows: TNFR1 (1 : 1000, Affinity, USA), NF-*κ*B P65 (1 : 1000, Cell Signaling Technology, USA), P-I*κ*B*α* (1 : 1000, Cell Signaling Technology, USA), ERK (1 : 1000, Affinity, USA), P-ERK (1 : 1000, Affinity, USA), JNK (1 : 1000, Cell Signaling Technology, USA), P-JNK (1 : 1000, Cell Signaling Technology, USA), MAPK P38 (1 : 1000, Cell Signaling Technology, USA), P-P38 (1 : 1000, Cell Signaling Technology, USA), iNOS (1 : 1000, Affinity, USA), Cox2 (1 : 1000, Affinity, USA), TNF-*α* (1 : 500, Affinity, USA), IL-6 (1 : 1000, Affinity, USA), IL-1*β* (1 : 1000, Affinity, USA), Desmin (1 : 1000, Affinity, USA), Vimentin (1 : 1000, Affinity, USA), and *α*-SMA (1 : 500, Affinity, USA).

### 2.9. Statistical Analysis

GraphPad Prism was used to perform Dunnett's multiple comparison test. The statistical analysis results were expressed as the mean value of SEM, and statistical analysis was analyzed by analysis of variance. *p* < 0.05 was considered statistically significant.

## 3. Result

### 3.1. Attenuating Effect of the Huangjia Ruangan Granule on CCl_4_ Liver Injury

**Figure 2:**
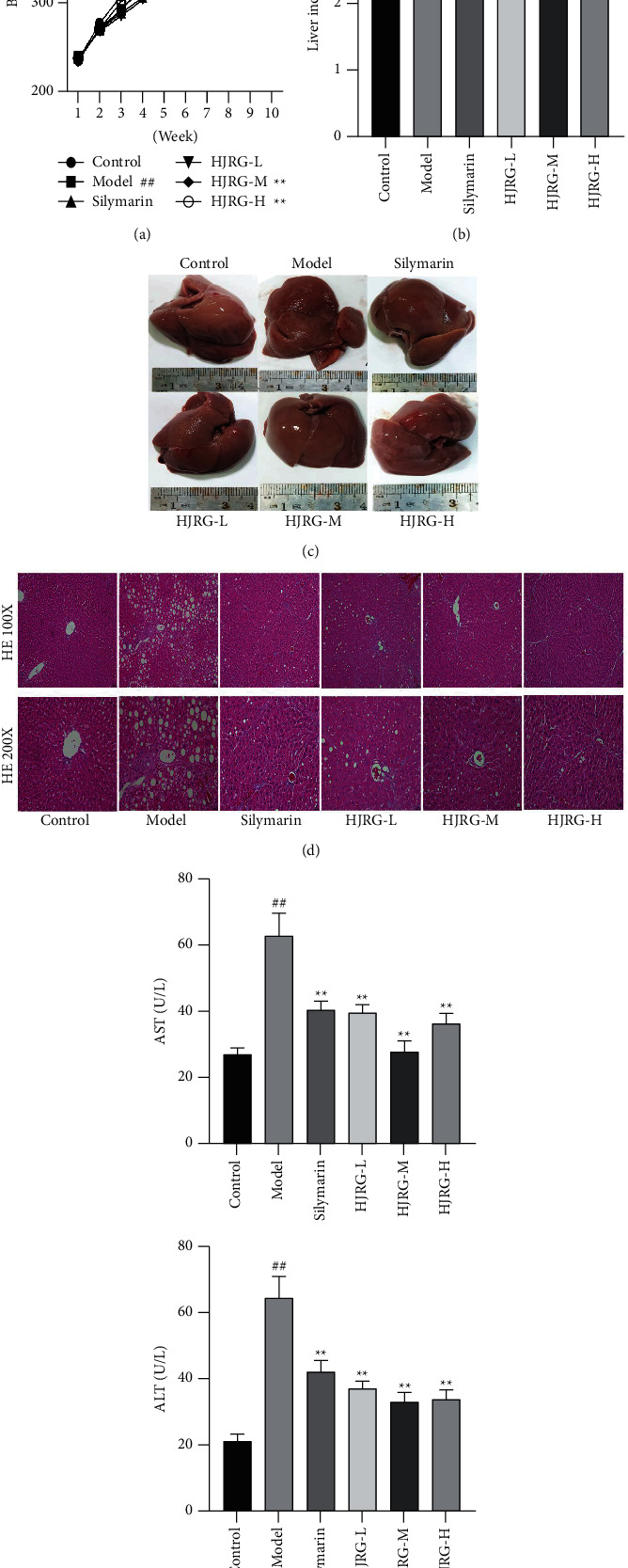
HJRG prevents the injury of CCl_4_ in rats. (a) Comparison of the body weight of rats in each group. (b) The Liver Index of rats in different groups (*n* = 12). (c) Appearance of the isolated liver of each group of rats. (d) HE staining of the liver tissue representative images (Magnification, 100X and 200X). (e) Concentrations of AST and ALT in the serum (*n* = 12). #*p* < 0.05 and ##*p* < 0.01, compared with the control group; ^*∗*^*p* < 0.05 and ^*∗∗*^*p* < 0.01, compared with the model group. Control: control group; Model: model group; Silymarin: silymarin group; HJRG-L: Huangjia Ruangan granule low-dose group; HJRG-M: Huangjia Ruangan granule medium-dose group; HJRG-H: Huangjia Ruangan granule high-dose group.

Compared with the control group, the weight of the model group increases slowly, and the last weight significantly decreased (Figure 2(a)). The Liver Index is significantly higher (Figure 2(b)), and the isolated liver is dim in color, with a rough and granular surface (Figure 2(c)). Compared with the model group, HJRG and silymarin can effectively alleviate the slow weight gain of rats, last weight gain, smooth surface, color of the liver, and significantly reduce the liver index of rats. Liver histopathological examination is the gold standard for the diagnosis of liver fibrosis. The histopathological changes in the liver tissue were detected in liver sections (Figure 2(d)). Evident changes were observed in the liver tissue of the model group, including necrosis, inflammatory cell infiltration, and diffuse fat changes. Compared with the model group, HJRG and silymarin treatment significantly ameliorated necrosis, inflammation, and steatosis. Serum AST and ALT activities are markers of liver toxicity [26]. Compared with the control group, the AST and ALT (Figure 2(e)) of the model group were significantly increased (*p* < 0.01). By contrast, HJRG and silymarin treatment significantly reduced serum AST and ALT activities. Therefore, the Huangjia Ruangan granule can regulate liver injury in rats.

### 3.2. Huangjia Ruangan Granule Improves Liver Fibrosis Induced by CCl_4_ in Rats

**Figure 3:**
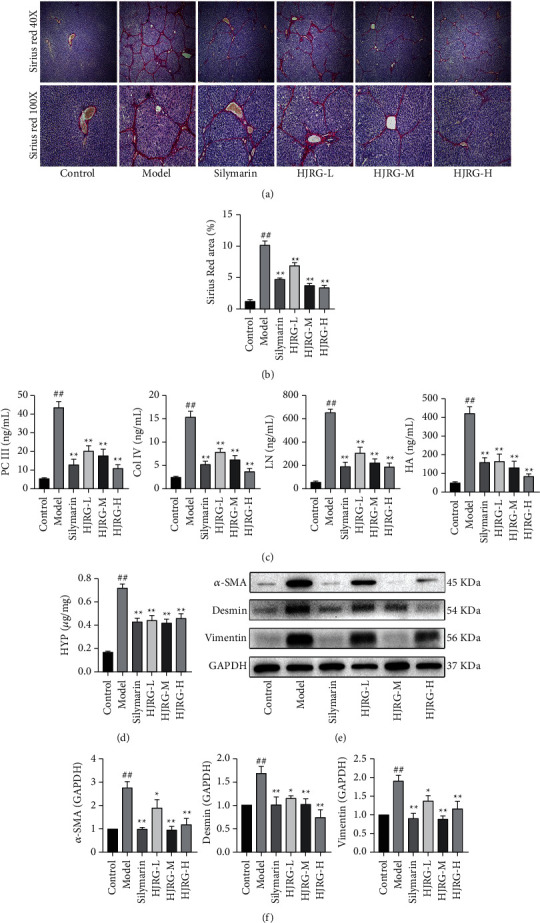
HJRG can reduce the progression of fibrosis. (a) Sirius red staining of liver tissue sections (magnification, 40X and 100X). (b) Quantification of collagen deposition by Sirius red staining (*n* = 3). (c) Four serum indexes of liver fibrosis: PC III, Col IV, LN, and HA (*n* = 12). (d) HYP contents in liver tissue (*n* = 8). (e) Effects of HJRG on protein levels of *α*-SMA, desmin, and vimentin in liver tissue. (f) Quantification of *α*-SMA, desmin, vimentin, and the protein expression in liver tissue (*n* = 5). #*p* < 0.05 and ##*p* < 0.01, compared with the control group; ^*∗*^*p* < 0.05 and ^*∗∗*^*p* < 0.01, compared with the model group. Control: control group; Model: model group; silymarin: silymarin group; HJRG-L: Huangjia Ruangan granule low-dose group; HJRG-M: Huangjia Ruangan granule medium-dose group; HJRG-H: Huangjia Ruangan granule high-dose group.

As shown in the figure (Figures 3(a)3(b)), the model group was stained with Sirius red to show the evidence of collagen accumulation, connective tissue deposition, and thin diaphragms formed between liver lobules, but under the treatment of HJRG and silymarin, the fibrous intervals, severity scores, and positive areas of collagen fibers in liver tissue were significantly reduced. PC III, Col IV, LN, and HA are considered important indicators of liver fibrosis [27], which can be used to reflect the extent of liver fibrosis. According to our results, PC III, Col IV, LN, and HA (Figure 3(c)) of the model group were significantly increased compared with those of the control group. By contrast, HJRG and silymarin treatments significantly reduced the serum PC III, Col IV, LN, and HA levels (*p* < 0.05). HYP is one of the main components of collagen tissue. The accumulation of HYP is significantly increased by collagen accumulation at the liver fibrosis sites and can be used to assess the extent of liver fibrosis [28]. The model group showed a high level of HYP content in the liver (*p* < 0.01) while the groups with HJRG and silymarin treatments reduced the HYP level in the liver (Figure 3(d)). *α*-smooth muscle actin (*α*-SMA) is known as a marker of HSC activation [29]. In the model group, the expression of *α*-SMA in the liver was significantly increased (Figures 3(e) and 3(f)), which indicated that HSC activated the myofibroblasts. Compared with the model group, HJRG and silymarin treatments significantly reduced the expression of *α*-SMA (*p* < 0.05), indicating that the myofibroblasts expressing *α*-SMA were inhibited. The measurement of liver desmin and vimentin indicated the production of collagen [30]. The contents of desmin and vimentin (Figures 3(e) and 3(f)) in the model group were significantly higher than those in the control group. HJRG and silymarin can reduce the desmin and vimentin content of liver fibrosis rats (*p* < 0.05). Therefore, HJRG treatment has a good effect on liver fibrosis indicators.

### 3.3. Network Pharmacological Analysis of Huangjia Ruangan Granule

**Figure 4:**
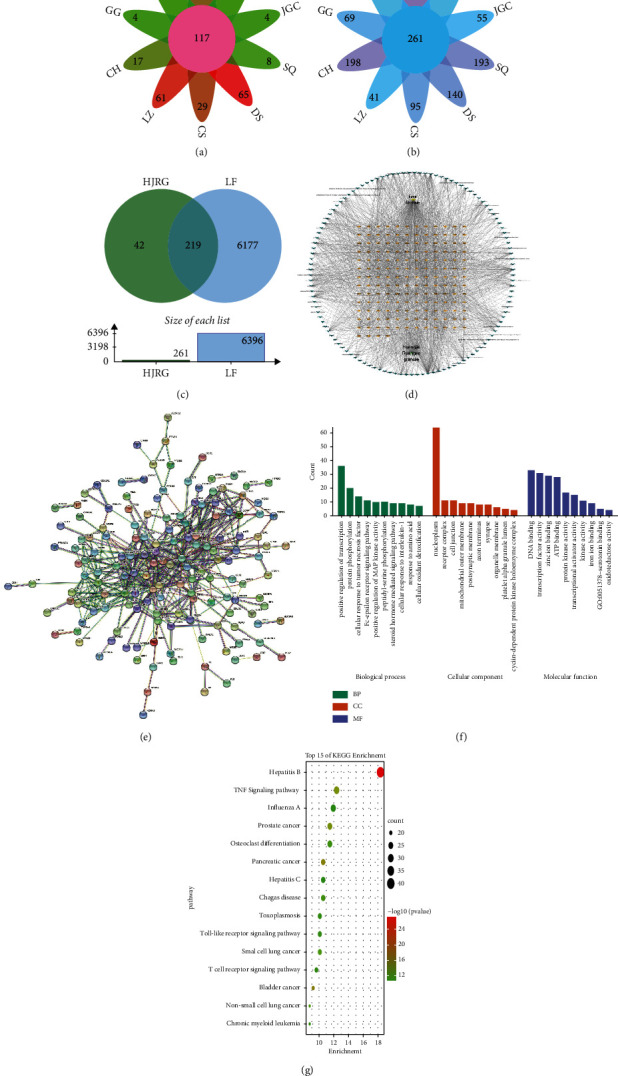
Analysis of network pharmacology related to HJRG. (a) The flower plot of the active ingredients in HJRG. The pink circle in the center represents the 117 active ingredients of HJRG. Each petal corresponds to the number of compounds in the corresponding Chinese medicine. (b) The flower plot of the potential target in HJRG. The pink circle in the center represents the 261 potential targets of HJRG. Each petal corresponds to the number of potential targets in the corresponding Chinese medicine. (c) A Venn diagram showing that HJRG shares 219 potential targets with the targets of liver fibrosis. (d) The component-target network. (e) PPI network analysis of potential targets. (f) Top 10 items of BP, CC, and MF in the GO enrichment analysis of potential targets. BP: biological process; CC: cellular component; MF: molecular function. (g) Top 15 items of the KEGG pathway analysis of the potential targets.

Based on the two standards (DL ≥ 0.18 and OB ≥ 30) [31], a total of 117 compounds have been identified in the TCM database. In particular, 20, 0, 4, 17, 61, 29, 65, 8, 4, and 0 candidate compounds were detected in HQ, BJ, GG, CH, LZ, CS, DS, SQ, JGC, and YXZ, respectively (Figure 4(a)). Then, we further explored the potential therapeutic targets. A total of 261 potential targets were found from the TCMSP and HIT databases (Figure 4(b)). Different components of HJRG have similar activities. This finding may be due to modulation with a shared target. We further searched 6396 liver fibrosis-related targets in the OMIM database, TTD database, GeneCards database, and DisGeNet database. The VENN diagram reflects that there are in total 219 shared targets between the Huangjia Ruangan granule and liver fibrosis-related targets (Figure 4(c)). In screening the core components and targets of HJRG, the C (component)-T (target) network constructed by Cytoscape was used (Figure 4(d), or Figure 1 in the supplemental files). These targets are candidate targets for HJRG with antiliver fibrosis activity. PPI network analysis was performed (Figure 4(e), or Figure 2 in the supplemental files). In addition, the Omicshare online tool was used to perform GO enrichment analysis (Figure 4(f)) and KEGG pathway analysis (Figure 4(g)) to determine the factors inhibiting liver fibrosis and to clarify the multitarget and multipathway mechanism of HJRG on liver fibrosis. Immune inflammation (IL-6 and NF-*κ*B) is a GO item that shows significant enrichment; in addition, the core target is the TNF signaling pathway.

### 3.4. Huangjia Ruangan Granule Improves Oxidative Stress in Rats with Liver Fibrosis

**Figure 5:**
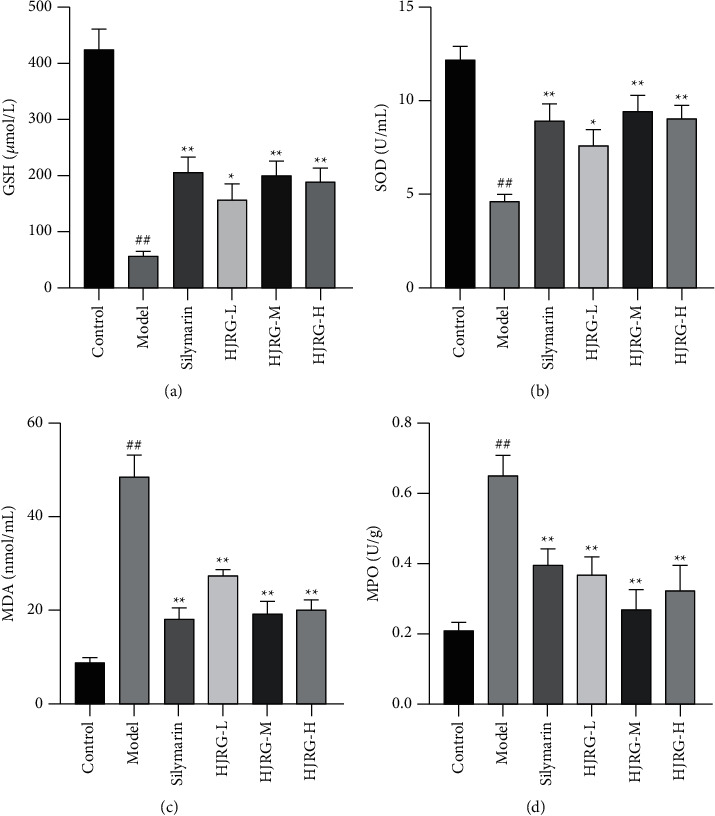
HJRG inhibited oxidative stress in rats. (a) Glutathione (GSH), (b) superoxide dismutase (SOD) activity, (c) malondialdehyde (MDA) content, and (d) myeloperoxidase (MPO) activity in the liver tissue. Data are presented as means ± SEM (*n* = 10 in each group). #*p* < 0.05 and ##*p* < 0.01, compared with the control group; ^*∗*^*p* < 0.05 and ^*∗∗*^*p* < 0.01, compared with the model group. Control: control group; Model: model group; Silymarin: silymarin group; HJRG-L: Huangjia Ruangan granule low-dose group; HJRG-M: Huangjia Ruangan granule medium-dose group; HJRG-H: Huangjia Ruangan granule high-dose group.

Liver damage is the final result of free-radical-mediated oxidative stress in the liver. CCl_4_ undergoes biotransformation in the liver to produce active metabolites, leading to the generation of a large number of free radicals. After the free radicals are formed, they will trigger a series of reactions and eventually lead to lipid peroxidation reactions [32]. Oxidative stress caused by free radicals is an important mechanism of liver damage induced by CCl_4_. SOD is an important antioxidant enzyme in the body, and GSH is the substrate for GSH-Px to decompose hydrogen peroxide [33]. Liver MPO activity is used as a marker of oxidative stress, inflammation, and tissue neutrophil accumulation and activation [34]. Our results showed that HJRG and silymarin had inhibitory effects on CCl_4_-induced oxidative stress damage in rats. Compared with the control group, the content of GSH (Figure 5(a)) and SOD (Figure 5(b)) in the model group decreased, and the content of MDA (Figure 5(c)) and MPO (Figure 5(d)) increased significantly. Compared with the model group, the administration of HJRG and silymarin significantly increased the content of SOD and GSH, and decreased the content of MDA and MPO. The comprehensive results indicate that HJRG may reduce liver fibrosis by alleviating the oxidative stress environment.

### 3.5. Huangjia Ruangan Granule Reduces Inflammation in Rats with Liver Fibrosis

**Figure 6:**
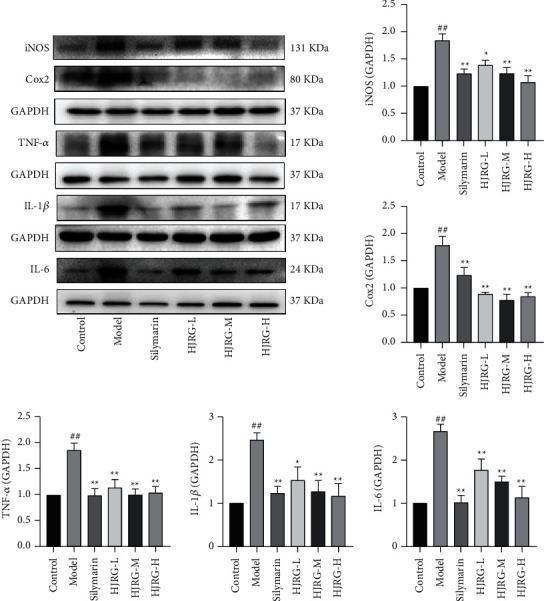
HJRG reduces inflammation in rats. Representative images of western blot analysis and the protein expression statistics of TNF-*α*, IL-1*β*, IL-6, Cox2, and iNOS in the liver. (*n* = 5). #*p* < 0.05 and ##*p* < 0.01, compared with the control group; ^*∗*^*p* < 0.05 and ^*∗∗*^*p* < 0.01, compared with the model group. Control: control group; Model: model group; Silymarin: silymarin group; HJRG-L: Huangjia Ruangan granule low-dose group; HJRG-M: Huangjia Ruangan granule medium-dose group; HJRG-H: Huangjia Ruangan granule high-dose group.

Inflammation usually leads to liver fibrosis, and the development of fibrosis is usually accompanied by an increase of inflammatory factors [35]. Therefore, we further evaluated the inhibitory effect of HJRG on inflammatory responses in the progression of fibrosis. Compared with the control group, the protein expression levels of TNF-*α*, IL-1*β*, IL-6, Cox2, and iNOS (Figure 6) in the liver of the model group were significantly upregulated (*p* < 0.05). Compared with the model group, the protein expression levels of TNF-*α*, IL-1*β*, IL-6, Cox2, and iNOS in the HJRG and silymarin treatment groups were significantly downregulated (*p* < 0.05). Our comprehensive results show that HJRG treatment has a great inhibitory effect on the inflammatory response induced by CCl_4_.

### 3.6. Huangjia Ruangan Granule Regulated TNF/MAPK and NF-*κ*B Signaling Pathways of Rats with Liver Fibrosis

**Figure 7:**
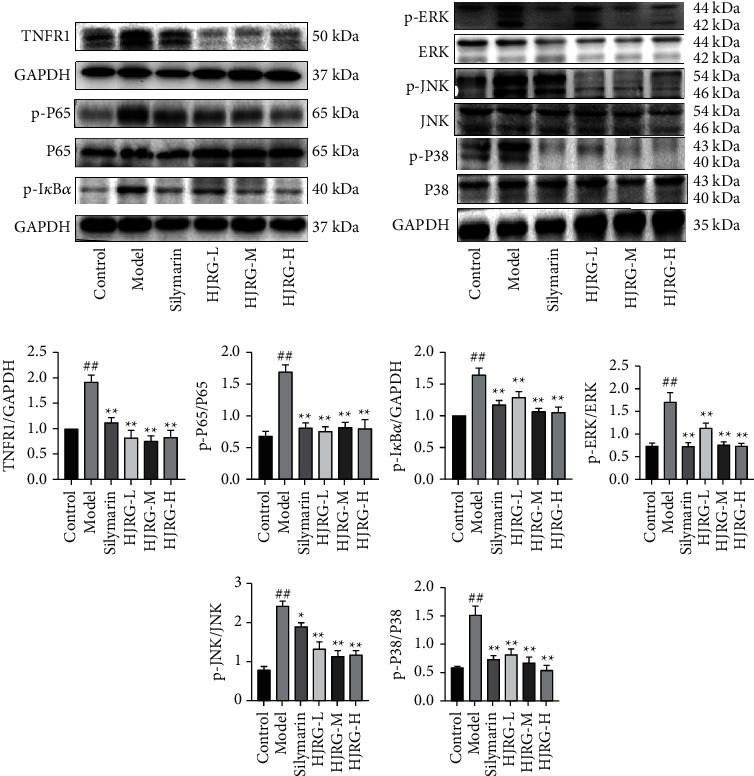
HJRG regulated TNF/MAPK and NF-*κ*B signaling pathways in rats. Representative images of western blot analysis and the protein expression statistics of TNFR1, p-I*κ*B*α*, p-P65/P65, p-ERK/ERK, p-JNK/JNK, and MAPK p-P38/P38 in the liver. (*n* = 5). #*p* < 0.05 and ##*p* < 0.01, compared with the control group; ^*∗*^*p* < 0.05 and ^*∗∗*^*p* < 0.01, compared with the model group. Control: control group; Model: model group; Silymarin: silymarin group; HJRG-L: Huangjia Ruangan granule low-dose group; HJRG-M: Huangjia Ruangan granule medium-dose group; HJRG-H: Huangjia Ruangan granule high-dose group.

To further clarify the antifibrosis mechanism of HJRG, we detected the expression of related proteins in TNF/MAPK and NF-*κ*B signaling pathways. As shown in Figure 7, compared with the control group, the protein expression of TNFR1, p-I*κ*B*α*, p-P65/P65, p-ERK/ERK, p-JNK/JNK, and MAPK p-P38/P38 in the liver tissue was markedly increased in the model group (*p* < 0.05). Compared with the model group, the protein expression levels of TNFR1, p-I*κ*B*α*, p-P65/P65, p-ERK/ERK, p-JNK/JNK, and MAPK p-P38/P38 in the HJRG and silymarin treatment groups was markedly decreased (*p* < 0.05). The results indicated that HJRG could inhibit the TNF/MAPK and NF-*κ*B signaling pathways.

## 4. Discussion

Liver fibrosis is a pathological process of abnormal liver connective tissue proliferation and a key step in the progression of chronic liver disease to cirrhosis. Its prognosis depends on whether it can block or reverse the progression of cirrhosis [36]. Therefore, in the early stages of hepatic fibrosis, the main treatment's main focus is on the etiological treatment and anti-inflammation hepatoprotection [37]. HJRG has the effects of relieving liver stagnation and depression and promoting blood circulation, which are important for intervening liver fibrosis and early cirrhosis. It has been effectively used in clinical practice in recent years. Evidence accumulated from animal and human studies shows that HJRG can reduce liver damage, especially by reversing liver fibrosis. However, the mechanism of HJRG's antiliver fibrosis remains unclear.

In this study, we established a liver fibrosis model in rats induced by CCl_4_ and intervened with the Huangjia Ruangan granule. Silymarin is the main active ingredient of *Silybum marianum*. It is the most studied botanical drug with important antioxidant properties [38]. It has been used to treat human liver cirrhosis, reverse fibrosis, and stimulate regeneration [39]. Therefore, we selected silymarin as the positive drug. Based on the results, HJRG can not only effectively restore liver tissue abnormalities, but also reduce the concentration of ALT and AST in the serum of rats with liver fibrosis induced by CCl_4_ and then prevent liver necrosis and inflammatory cell infiltration, indicating that HJRG can relieve liver damage during fibrosis.

The characteristic of liver fibrosis is due to the imbalance between ECM production and degradation, resulting in excessive deposition of ECM in the liver [40]. The activation of HSC has been identified as a key event in the development of liver fibrosis, which regulates the mass production and secretion of ECM [41]. In this study, HJRG can effectively inhibit collagen accumulation and HYP contents in liver tissues; reduce serum PC III, Col IV, LN, and HA levels, and the overexpression of *α*-SMA, desmin, and vimentin in the liver tissue; inhibit the activation of HSC and ECM deposition in the liver, thereby inhibiting liver fibrosis.

The overall process of liver fibrosis is complex, and achieving an appropriate therapeutic effect on a single target is difficult. As a traditional Chinese medicine prescription, HJRG is involved in multiple levels and targets and focuses on the overall regulation and treatment of liver fibrosis. Thus, we checked the candidate components and targets of HJRG using the network pharmacological analysis and found 117 candidate compounds and 219 targets corresponding to antiliver fibrosis. Further GO enrichment analysis and KEGG pathway analysis of candidate targets showed that HJRG primarily regulated inflammation through the TNF signaling pathway and improves oxidative stress antiliver fibrosis effects.

Oxidative stress is the main pathogenesis of CCl_4_-induced liver fibrosis in rats [42]. The toxicity of CCl_4_ is mediated by metabolic activation, which leads to peroxidation in the body by generating highly active trichloromethyl free radicals [43]. Results showed that the level of reduced GSH in the liver tissue of the model group was significantly decreased. GSH is a major antioxidant. Glutathione reductase resists oxidative stress and protects macromolecules and cell membranes from free radical damage [44]. The examination of liver biochemical markers also shows that a large number of free radicals produced by CCl_4_ interference include SOD, MDA, and MPO. SOD provides the main defense against oxidative stress by scavenging free radicals. MDA, and MPO levels reflect the oxidative damage that causes liver tissue damage [45]. However, after the administration of HJRG, the levels of GSH and SOD can be significantly restored, and the activity of MDA and MPO can be reduced, helping the body to scavenge free radicals generated by the CCl_4_ stress and self-protect against the oxidative stress. Therefore, HJRG can alleviate liver fibrosis through antioxidant effects.

Hepatic immune-inflammatory mechanisms are essential to maintain liver homeostasis. Evidence shows that CCl_4_ toxicity can trigger the recruitment inflammatory mediators, such as TNF-*α*, IL-1*β*, IL-6, Cox2, and iNOS, thereby inducing liver inflammation [46]. TNF-*α*, IL-1*β*, and IL-6 mainly contribute to HSCs activation; Cox2 and iNOS are the main regulators of the inflammatory response; Cox2 induces prostaglandin E2 (PGE2) production and then mediates inflammation in the liver disease models [47]; the continuous production of NO is associated with the increased iNOS expression. In our experiment, HJRG downregulated the expression of the proinflammatory factors (TNF-*α*, IL-1*β*, and IL-6). In addition, HJRG downregulated the expression of Cox2 and iNOS in the liver tissue. That is, HJRG can prevent the recruitment of inflammatory mediators of liver fibrosis, thereby improving liver fibrosis.

**Figure 8:**
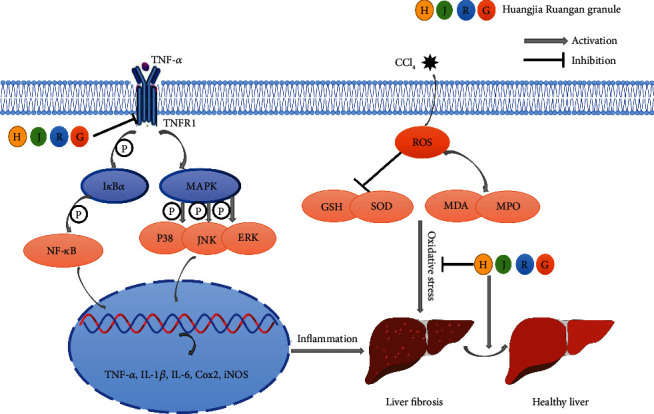
Schematic diagram of the proposed mechanisms underlying the protective effects of HJRG inhibiting CCl_4_-induced liver fibrosis in rats. The mechanism of HJRG may be inhibiting the TNFR1-mediated MAPK and NF-*κ*B signaling pathways, reducing the inflammatory response, and ameliorating oxidative stress, thereby inhibiting liver fibrosis.

Our study identified the TNF signaling pathway as the main signal pathway in the treatment of liver fibrosis with HJRG. Upregulation of TNFR1, one of the TNF signaling receptors, was correlated with incident liver fibrosis [48]. Evidence shows that TNFR1 activates the downstream MAPK signaling pathways, including ERK, JNK, and MAPK P38 pathways [49]. On the other hand, TNFR1 can also induce the phosphorylation of I*κ*B*α*, which promotes the phosphorylation of NF-*κ*B P65 to participate in the inflammatory reaction together with the MAPK signaling pathway, regulating the process of liver fibrosis [50]. Our study found that HJRG significantly inhibited the increase of p-I*κ*B*α*, p-P65/P65, p-ERK/ERK, p-JNK/JNK, and MAPK p-P38/P38 ratios in the liver tissue of rats with hepatic fibrosis and the protein expression levels of TNFR1. This study demonstrates that HJRG may exert antifibrotic effects by inhibiting the activation of TNFR1 and the downstream MAPK and NF-*κ*B pathways (Figure 8).

## 5. Conclusions

Our research proved that HJRG can effectively reduce the liver damage and oxidative stress caused by CCl_4_, and the mechanism may be the inhibition of TNFR1-mediated MAPK and NF-*κ*B signaling pathways to reduce the inflammatory response, thereby inhibiting liver fibrosis. The present study may provide experimental evidence for the study and usage of HJRG in liver fibrosis.

## Data Availability

The datasets analyzed in this article are publicly available, and further inquiries can be directed to QC (1367084356@qq.com).
