# Supplementary figures and images for: Huangjia Ruangan Granule Inhibits Inflammation in a Rat Model with Liver Fibrosis by Regulating TNF/MAPK and NF-κB Signaling Pathways
Source: Evid Based Complement Alternat Med. 2022 Jul 30;2022:8105306. doi: 10.1155/2022/8105306 (PMC9356785; doi:10.1155/2022/8105306)

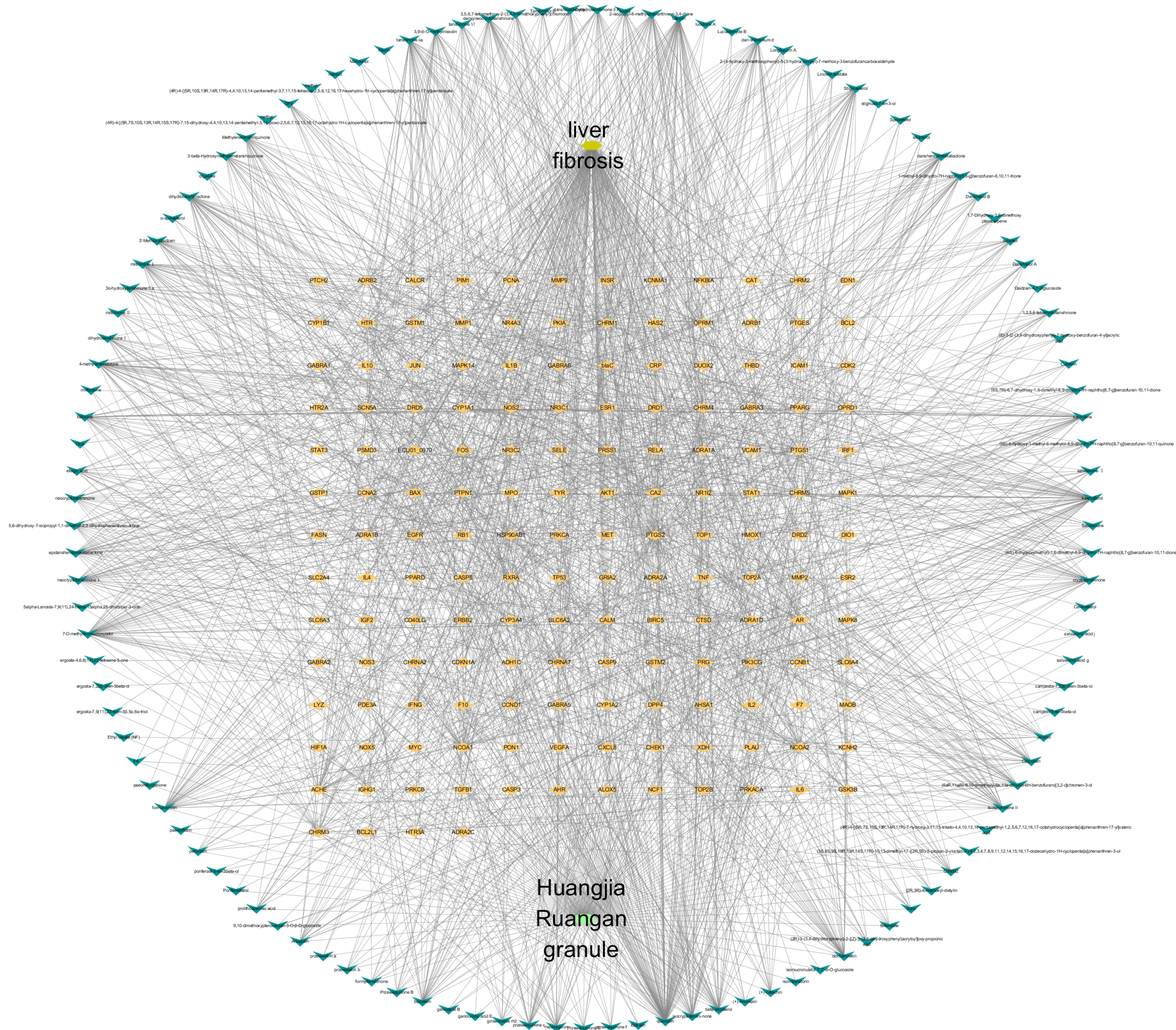

Supplement: Supplementary Materials — Figure 4(d) can be found in Figure 1 in the supplemental files, and Figure 4(e) can be found in Figure 2 in the supplemental files. [file 8105306.f1.zip › 8105306.f1/supplementary figure 1(1).pdf]

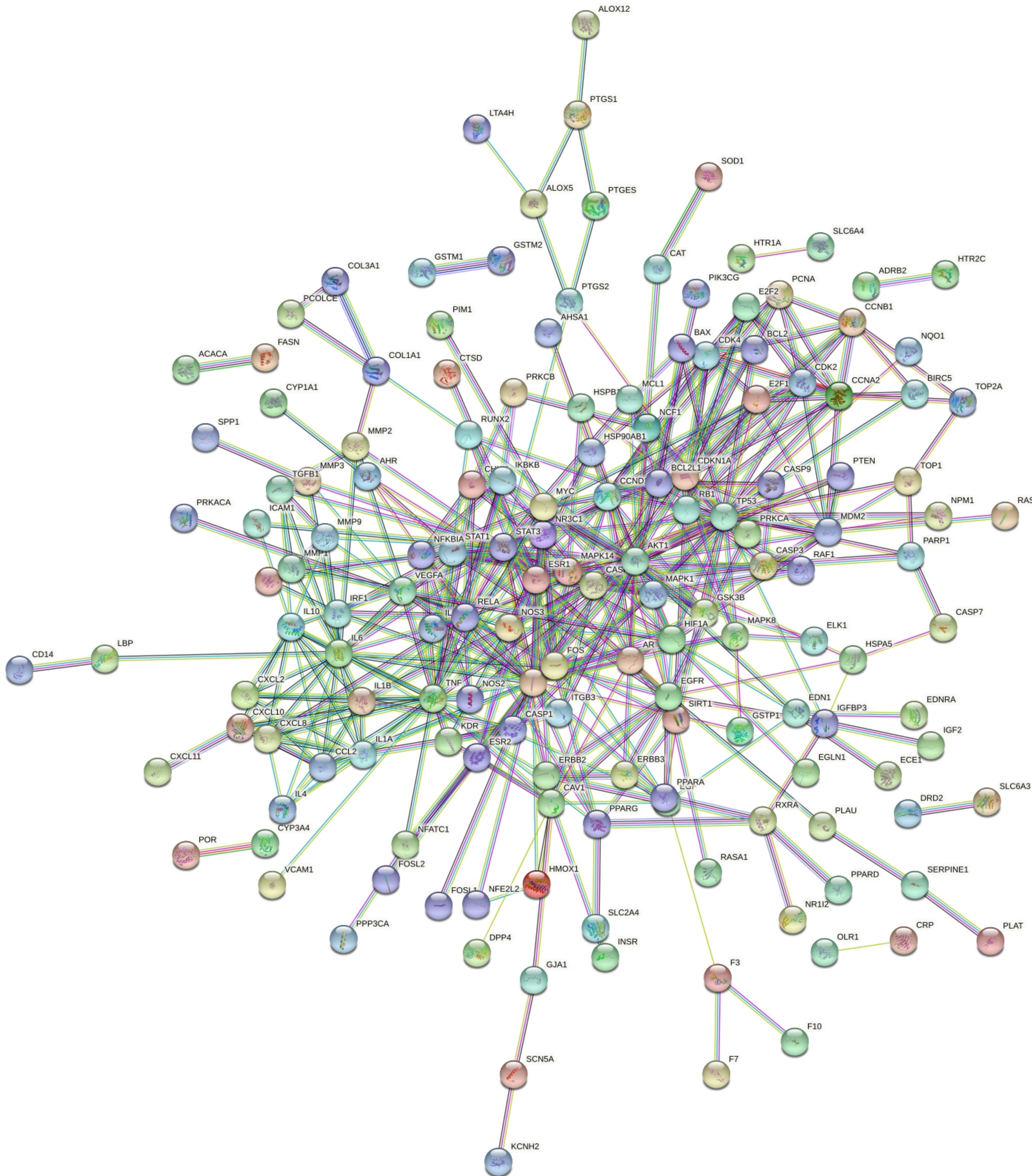

Supplement: Supplementary Materials — Figure 4(d) can be found in Figure 1 in the supplemental files, and Figure 4(e) can be found in Figure 2 in the supplemental files. [file 8105306.f1.zip › 8105306.f1/supplementary figure 2.pdf]
